# Metformin Improves Stemness of Human Adipose-Derived Stem Cells by Downmodulation of Mechanistic Target of Rapamycin (mTOR) and Extracellular Signal-Regulated Kinase (ERK) Signaling

**DOI:** 10.3390/biomedicines9121782

**Published:** 2021-11-27

**Authors:** Somaiah Chinnapaka, Katherine S. Yang, Quinn Flowers, Minhal Faisal, Wayne Vincent Nerone, Joseph Peter Rubin, Asim Ejaz

**Affiliations:** Department of Plastic Surgery, University of Pittsburgh, Pittsburgh, PA 15261, USA; chinnapakas@upmc.edu (S.C.); KSY6@pitt.edu (K.S.Y.); quinnflowers@ucsb.edu (Q.F.); MIF65@pitt.edu (M.F.); neronewv@upmc.edu (W.V.N.); rubipj@upmc.edu (J.P.R.)

**Keywords:** adipose stem cells, adipose tissue, stemness, differentiation, proliferation, metformin, mTOR, ERK, autophagy

## Abstract

Adipose tissue plays an important role in regulating metabolic homeostasis by storing excess fat and protecting other organs from lipotoxicity. Aging is associated with central fat redistribution, culminating in a decrease in insulin-sensitive subcutaneous and an increase in insulin-resistant visceral adipose depots. Adipose-derived stem cells (ASCs) play an important role in the regeneration of adipose tissue. Aged ASCs show decreased stemness and regenerative potential due to the accumulation of oxidative stress and mitochondrial dysfunction-related cell damage. Metformin is a well-established anti-diabetic drug that has shown anti-aging effects in different organisms and animal models. In this study, we analyzed the effect of metformin treatment on the stemness of human ASCs in cell culture and whole adipose tissue culture models. Our results demonstrate that metformin improves the stemness of ASCs, reducing their rate of proliferation and adipocyte differentiation. Investigating the possible underlying mechanism, we observed a decrease in the mTOR and ERK activity in metformin-treated ASCs. In addition, we observed an increase in autophagy activity upon metformin treatment. We conclude that metformin treatment improves ASCs stemness by reducing mTOR and ERK signaling and enhancing autophagy. Future in vivo evaluations in animal models and humans will pave the way for the clinical adaptation of this well-established drug for reviving the stemness of aged stem cells.

## 1. Introduction

Adipose tissue is a dynamic organ that contributes an important homeostatic role in regulating nutrient balance, insulin sensitivity, and immune modulation. Adipose tissue performs this role by storing and oxidizing excess fatty acids, thus preventing steatosis and lipotoxicity in other organs [1]. Age-associated insulin resistance is the most prevalent metabolic disease. Approximately 25% of the U.S. population over 65 suffers from diabetes, resulting in higher mortality rates, reduced functional status, increased risk of hospitalization, and excessive burden on the health economy [2]. Although the association between diabetes and obesity has long been recognized, the link between aging and type 2 diabetes remains elusive [3]. The first observation relating decline in glucose tolerance to aging was made in 1921 [4]. Several studies have since identified reduced insulin sensitivity as the primary cause of the age-related impairment of glucose metabolism [5,6]. Based on anatomical localization, adipose tissue can be broadly subdivided into two depots: subcutaneous and visceral adipose depots. The location and function of the adipose tissue depot are important in terms of insulin sensitivity; for example, the visceral depot is more resistant whereas the subcutaneous depot is more sensitive to insulin [7]. An age-related gradual loss of subcutaneous adipose tissue volume results in reduced glucose and lipid uptake, leading to ‘lipid overflow’ and ectopic deposition of lipids in the muscle and liver, contributing to the development of insulin resistance [8].

Adipose tissue is composed of different cell types. Enzymatic digestion of adipose tissue with collagenase produces two main fractions: the adipocyte fraction and the stromal vascular fraction (SVF) [9]. SVF comprises adipose-derived stem cells (ASCs), lymphocytes, endothelial cells, pericytes, and fibroblasts [10]. ASCs are defined immune-phenotypically as CD34^+^CD90^+^CD29^+^CD45^-^CD31^-^ cells of mesenchymal origin, exhibiting tri-lineage differentiation capabilities into bone, cartilage, or fat [11,12]. In white adipose tissues, these cells mainly reside in the vascular stroma around small blood vessels and can proliferate and differentiate into adipocytes [11]. Adipose-derived stem cells (ASCs) play an important role in the regeneration of adipose tissue by undergoing proliferation, self-renewal, and differentiation. Aging is associated with loss of these stemness properties in ASCs [13,14]. An age-related decline in subcutaneous fat depot size is believed to be due to altered replication and differentiation of ASCs [15,16,17]. Findeisen et al. suggest accumulation of oxidative stress in adipose tissue during aging, which negatively impacts the differentiation capacity; [15] however, the exact mechanism(s) underlying the loss of stemness in ASCs is not understood. Recent studies have shown that reduced autophagy, higher mechanistic target of rapamycin (mTOR) pathway activity, and accumulation of reactive oxygen species (ROS) are linked to loss of stemness in ASCs and premature senescence induction in ASCs [18,19,20].

Metformin is an anti-diabetic drug commonly used to lower blood sugar and improve insulin sensitivity. It can be used to treat a variety of aging-related metabolic disorders, like cardiovascular disease, cancers, and cognitive decline [21,22]. The longevity-enhancing effects of metformin have been demonstrated in worms, mice, and rats through dietary restriction, which mimics the effect of stimulation of adenosine monophosphate-activated protein kinase activity (AMPK) [23]. Various studies have shown that metformin inhibits the mammalian target of rapamycin (mTOR) [24,25]. AMPK-mTOR signaling pathways specifically regulate the characteristics of cellular homeostasis, such as autophagy, proliferation, energy metabolism, and protein synthesis [26]. Metformin has been shown to inhibit proliferation, differentiation, and inflammation, reducing oxidative stress and mitochondrial potential and improving overall stemness in different types of adult stem cells [27,28]. The effect of metformin on the metabolism and stemness of ASCs in vitro and in vivo is not clear.

In this study, we employed 2D culture of human ASCs and the whole human adipose tissue culture techniques to simulate the in vivo cellular organization and tissue microenvironment. Using these experimental models, we studied the effect of metformin treatment on the differentiation, proliferation, and stemness of ASCs and investigated the molecular mechanisms involved in these cellular processes.

## 2. Materials and Methods

### 2.1. Donor Specifications

Adipose tissue was collected from five non-diabetic female donors in the age range 39 ± 13 and BMI range 27 ± 4 undergoing elective plastic surgery procedures at the University of Pittsburgh Medical Center (UPMC). The procedure of tissue collection was approved by the University of Pittsburgh Institutional Review Board (IRB No. 0511186).

### 2.2. Isolation and Cultivation of Human ASCs

The adipose precursor cells were isolated as follows [19,29]. Adipose tissue biopsies after surgery procedures were transferred to the lab in a sterile sealed container. Tissue was rinsed with Dulbecco’s phosphate-buffered saline (PBS; Sigma-Aldrich, St. Louis, MO, USA) followed by removal of fibrous material and blood vessels by dissection. The tissue was cut into pieces (1–2 mg) and digested in digestion buffer (HBSS containing 200 U/mL collagenase (CLS Type II, Worthington Biochemical Corp., Lakewood, NJ, USA) and 2% *w/v* BSA (Sigma-Aldrich, St. Louis, MO, USA)) under stirring for 60 min at 37 °C; 1 mg adipose tissue/3 mL digestion buffer. The dispersed tissue was centrifuged for 10 min at 200× *g* at room temperature. The floating adipocytes were aspirated, and the pelleted cells of the stromal vascular fraction (SVF) were suspended in erythrocyte lysis buffer (0.155 M NH4CI, 5.7 mM K2HPO4, 0.1 mM EDTA, pH 7.3) and incubated for 10 min at room temperature. To remove tissue debris, the cell suspension was filtered through a nylon mesh (pore size 100 μm; Thermo Fisher Scientific, Waltham, MA, USA). After another centrifugation step (10 min at 200× *g*) the pelleted SVF was suspended in ASC medium (Dulbecco’s modified Eagle’s medium (DMEM)/F-12 medium (1:1) with HEPES and L-glutamine (Thermo Fisher Scientific, Waltham, MA, USA), supplemented with 33 μM biotin (Sigma-Aldrich, St. Louis, MO, USA), 17 μM pantothenate (Sigma-Aldrich, St. Louis, MO, USA), 10 ng/mL EGF, 1 ng/mL bFGF, 500 ng/mL insulin, 2.5% fetal bovine serum, and 12.5 μM/mL gentamicin (Sigma-Aldrich, St. Louis, MO, USA) and seeded at a density of 50,000 cells/cm^2^ in T175 flasks.

After reaching 70% confluence, the cells were washed with PBS and trypsinized using 0.05% trypsin-EDTA 1x solution (Sigma-Aldrich, St. Louis, MO, USA). Trypsin was inactivated by the addition of ASC medium plus 10% FBS and removed by centrifugation at 300× *g* for 5 min before the cells were seeded in a density of 5000 cells/cm^2^. ASCs were again grown to 70% confluence before splitting. ASCs in passages 3–4 were used in this study. Cells were treated with different concentrations of metformin (Sigma-Aldrich, St. Louis, MO, USA), rapamycin (Sigma-Aldrich, St. Louis, MO, USA), or U0126 inhibitor according to the experimental scheme.

### 2.3. Adipogenic Differentiation

For the induction of adipogenesis, the ASCs were seeded at a density of 50,000 cells/cm^2^ in 6-well cell culture plates. Adipogenesis was induced using differentiation medium, composed of 0.2 μM insulin (Sigma-Aldrich, St. Louis, MO, USA), 0.5 mM 1-methyl-3-isobutylxanthine (IBMX) (Sigma-Aldrich, St. Louis, MO, USA), 0.25 μM dexamethasone (Sigma-Aldrich, St. Louis, MO, USA), and 10 μg/mL transferrin (Sigma-Aldrich, St. Louis, MO, USA) in ASC medium. After 3 days of differentiation, the medium was changed, and the cells were cultivated in the differentiation medium without IBMX for 14 days.

### 2.4. Adipose Tissue Culture

Adipose tissue culture was performed in 6-well cell culture plates, with some modification, as published by Harms et al. [30]. Whole adipose tissue was chopped into smaller pieces 3–5 mm in size and cultured in 5 mL cell culture media. Cell strainers with pore size of 70 µM were kept over the tissue fragments to keep the tissue submerged in media. The tissue fragments were digested with collagenase, as explained above for the ASCs isolation procedure, and the SVF was analyzed for expression of ASC-associated stemness genes.

### 2.5. Oil Red O Staining

For visualization of lipid droplets, cells were fixed with 4% paraformaldehyde in PBS for 1 h and stained with 0.3% Oil Red O (Sigma-Aldrich, St. Louis, MO, USA) in isopropanol/water (60:40) for 1 h. Final washing was carried out twice with distilled water. For quantification of absorbed Oil Red O, the stain was eluted with isopropanol (Sigma-Aldrich, St. Louis, MO, USA), and optical density was measured at 518 nm.

### 2.6. Bodipy Staining

On day 14 post-induction of adipogenesis, cells were stained with Bodipy (Invitrogen, Waltham, MA, USA) and Hoechst (Sigma-Aldrich, St. Louis, MO, USA) for 30 min at 37 °C. Images were acquired using the fluorescent microscope and analyzed with the ImageJ software.

### 2.7. Flowcytometry

ASCs in passage three were used for flowcytometric analyses for the surface expression of CD29 (Biolegend, San Diego, CA, USA), CD90 (Biolegend, San Diego, CA, USA), CD45 (BD, Franklin Lakes, NJ, USA), CD24 (Biolegend, San Diego, CA, USA), and CD31 (BD, NJ, USA). Cells were trypsinized, washed with PBS, and stained with antibodies in FACS staining buffer (1%BSA in PBS). Unstained ASCs were employed as a control. Fluorescent signal was measured using a flow cytometer (Fortessa, BD, Franklin Lakes, NJ, USA), and data were analyzed using FlowJo software (BD, Franklin Lakes, NJ, USA).

### 2.8. Osteogenic Differentiation

ASCs were grown to confluence in 6-well plates, and osteogenic differentiation was induced using osteogenic medium (DMEM 10%FBS, 0.1µM dexamethasone, 10 mM β-glycerol phosphate, and 50 μM Ascorbic Acid). At day 21 post-differentiation, cells were fixed in 4% paraformaldehyde (Sigma-Aldrich, St. Louis, MO, USA) and stained with Alizarin Red Stain (Sigma-Aldrich, St. Louis, MO, USA). Quantification of staining was performed using Image J software.

### 2.9. Apoptosis Detection by Annexin V-APC/PI Staining

Apoptotic cell death was measured using an Annexin V-APC/PI kit (Biolegend, San Diego, CA, USA). A total of 10,000 (cells/well) ASCs were seeded onto 6-well plates and incubated at 37 °C with 5% CO_2_. The cells were treated with metformin (0.25 mM, 0.5 mM, 1 mM, 2.5 mM, and 5 mM) (Sigma-Aldrich, St. Louis, MO, USA). After 72 h of treatment, the cells were collected by trypsinization and stained with 2.5 μL AnnexinV-APC and 2.5 μL PI in binding buffer, incubated in the dark at room temperature for 15 to 20 min, and analyzed by flow cytometry (Fortessa, BD, NJ, USA).

### 2.10. Reactive Oxygen Species Detection

ROS levels were determined using fluorescent dye 2′,7′-dichlorofluorescin diacetate (DCFH2-DA) probe (Invitrogen, Waltham, MA, USA). ASCs (10,000 cells/well) were cultured in a 6-well plate and incubated with metformin (5 mM) at 37 °C, 5% CO_2_ for 72 h. After incubation, ASCs were trypsinized and stained with 50μM DCFH2-DA dye for 30 min at 37 °C and analyzed using a fluorescence microscope.

### 2.11. TMRM Staining

ASCs (10,000 cells/well) were cultured in a 6-well plate, treated with different doses of metformin (5 mM) for 72 h, and incubated at 37 °C with 100 nM TMRM (Sigma-Aldrich, St. Louis, MO, USA) for 30 min at 37 °C. After incubation, cells were observed under a fluorescence microscope. Image J was used to quantify the fluorescence of microscopic images.

### 2.12. Quantitative RT-PCR Gene Expression

Total RNA was isolated with the RNeasy Micro Kit (Qiagen, Hilden, Germany), and cDNA synthesis was performed with the RevertAid First Strand cDNA Synthesis Kit (Thermo Fisher Scientific, Waltham, MA, USA). Gene-specific primers were purchased from Sigma-Aldrich, St. Louis, MO, USA. Quantitative expression analysis was performed using Quantstudio 3 (Thermo Fisher Scientific, Waltham, MA, USA). The mRNA quantification was performed using β-actin for normalization. Data for each gene transcript were normalized by calculating the difference (∆Ct) from the Ct-housekeeping and Ct-Target genes. The relative increase or decrease in expression was calculated by comparing the reference gene with the target gene (∆∆Ct) using the formula for relative expression (=2^∆∆Ct^).

### 2.13. Western Blot

Western blot analysis was performed essentially as described [19]. The method used to normalize the protein levels was “Pierce BCA Protein Assay Kit” (Thermo Fisher Scientific, Waltham, MA, USA). Cell lysates (15 μg total protein per lane) were prepared in sodium dodecyl sulfate (SDS) sample buffer, separated by SDS-PAGE, and blotted on polyvinylidene difluoride membranes. The following antibodies were used: mouse anti-human β-actin (Proteintech, IL, USA), perilipin (Cell Signaling, MA, USA), phosphor-P70S6K (Cell Signaling, MA, USA), total P70S6K (Cell Signaling, MA, USA), pERK1/2 (Cell Signaling, MA, USA), total ERK1/2 (Cell Signaling, MA, USA), anti-mouse IgG HRP conjugate (Proteintech, IL, USA), and rabbit anti-rat IgG HRP (Proteintech, IL, USA). Image J software was used for densitometric analyses.

### 2.14. Statistical Analysis

Statistical analyses were performed in GraphPad Prism (GraphPad Software Inc., La Jolla, CA, USA). FlowJo was used for FACS analysis. The significance of the difference between means was assessed by the Student’s t-test or analysis of variance. Error bars are represented as the mean ± SEM. Values were significant at *p* values of <0.05 (* *p* < 0.01 and # *p* < 0.05).

## 3. Results

**Figure 1:**
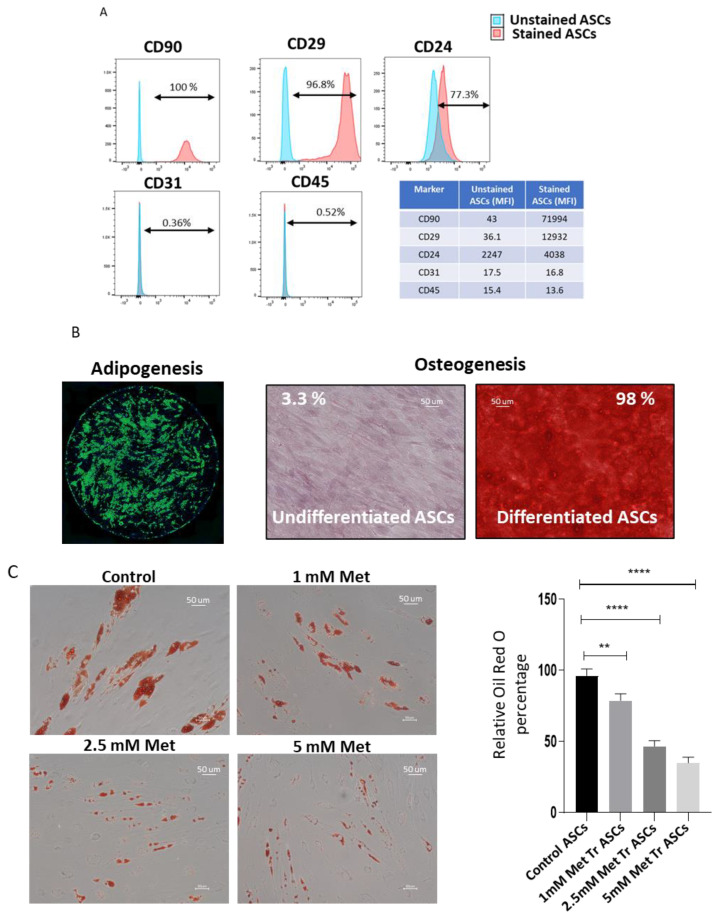

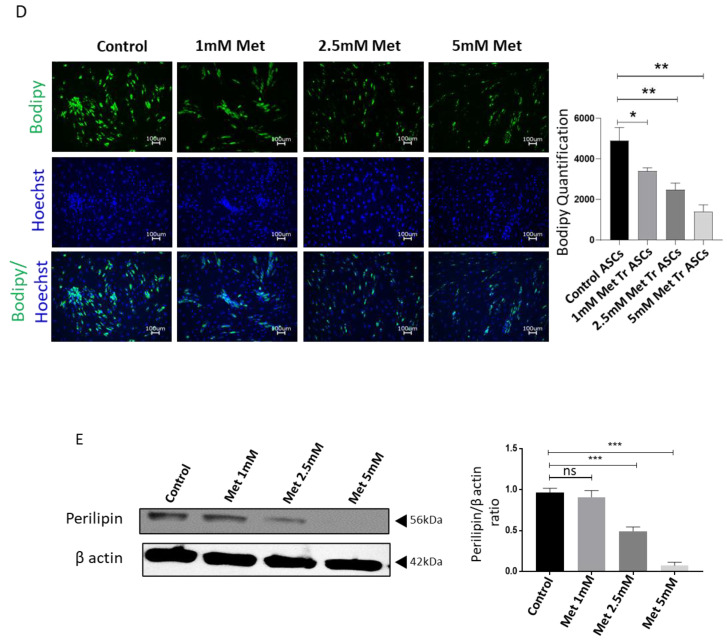
Metformin treatment inhibits adipogenic differentiation of ASCs in a dose-dependent manner. (**A**) ASCs were tested for the expression of CD29, CD90, CD24, CD31, and CD45 by flow cytometry. Both percentage positivity shown in the histograms and mean fluorescence intensity (MFI) shown in the table of control (unstained ASCs) and stained (ASCs treated with respective antibodies) are presented. (**B**) ASCs were subjected to adipogenic or osteogenic differentiation using lineage-specific differentiation cocktails. Differentiation to adipocyte lineage was confirmed by Bodipy staining, and osteogenesis was confirmed by Alizarin Red staining. The percentage of the Alizarin Red stained area was calculated using Image J. (**C**) ASCs were treated with an adipocyte differentiation cocktail in the presence or absence of different doses of metformin for 14 days. The development of adipocytes at day 14 post-induction was assessed by staining lipid droplets using Oil Red O stain. Absorbed Oil Red O stain was extracted, and optical density was measured. Optical density from control ASCs was taken as 100 percent, and relative change upon metformin treatment was plotted (*n* = 3). (**D**) Lipid content at day14 was determined using Bodipy stain (Green). Hoechst (Blue) was used to stain nuclei. (**E**) Western blot analysis of perilipin protein expression. β-Actin was employed as the loading control. Quantification of perilipin protein band densities normalized to β-Actin used Image J. Images and graphs are representatives of three independent replicates (*n* = 3). * *p* < 0.05, ** *p* < 0.01, *** *p* < 0.001 and **** *p* < 0.0001 as compared with control.

Metformin is a well-established anti-diabetic drug that has been shown to play an important role in extending longevity [31]. To study the effect of metformin treatment on the stemness of adipose-derived stem cells, we isolated ASCs from adipose tissue of human donors by collagenase digestion. The isolated ASCs were plastic adherent and exhibited a fibroblastic or spindle-shaped morphology. Flow cytometry analysis showed that ASCs were positive for mesenchymal stem cell markers, such as CD29, CD90, and CD24, but negative for hematopoietic lineage marker CD45 and the endothelial lineage marker CD31 (Figure 1A). In addition to displaying the typical ASC phenotype, the ASCs showed robust adipogenic and osteogenic differentiation potential, identified by Bodipy and Alizarin Red S staining, respectively (Figure 1B). The characterized ASCs were used for further experiments in our study.

It has been reported in the literature that metformin reduces the adipogenic differentiation of stem cells. We aim to explore the regulation of adipocyte differentiation of ASCs upon metformin treatment. ASCs were treated with 1, 2.5, and 5 mM metformin starting 2 days before adipogenic induction and throughout the differentiation process. Bodipy and Oil Red O stains were used to visualize the oil droplets at day 14 post-induction of differentiation. The results of Oil Red O (Figure 1C) and Bodipy staining (Figure 1D) reveal that metformin treatment significantly inhibits adipogenic differentiation in a dose-dependent manner compared to control cells. The inhibition of adipogenic differentiation was further confirmed using Western blot analysis of adipocyte differentiation marker protein perilipin. For this, cell lysates were collected from differentiated cells at day 14 of adipogenic differentiation, and perilipin expression was analyzed. Our Western blot results show significant downregulation of perilipin expression upon metformin treatment during adipogenic differentiation (Figure 1E), which correlated with the decrease in Bodipy and Oil Red O stains. We conclude that metformin treatment inhibits the adipogenic differentiation of human ASCs, with the magnitude of inhibition positively correlating with the metformin treatment dose.

**Figure 2:**
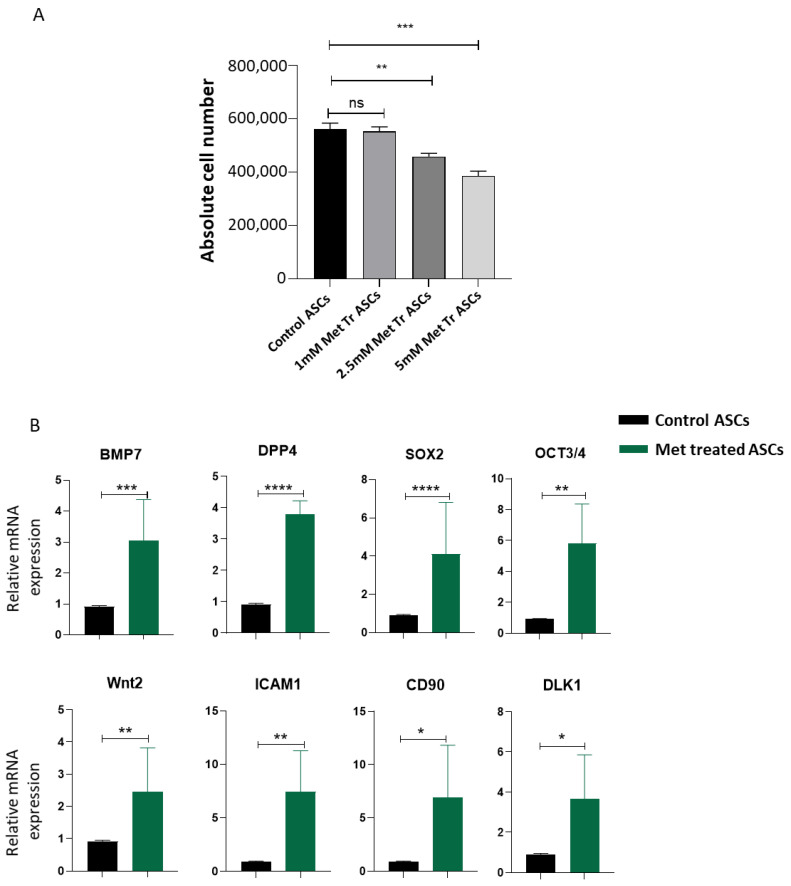
Metformin treatment reduces proliferation and promotes stemness-related gene expression in ASCs. (**A**) ASCs were treated with varying doses of metformin for 4 days. At the end of treatment duration, cells were trypsinized, washed with PBS, and resuspended in an equal volume of PBS. Cells were diluted 1:1 with trypan blue dye, counted using Improved Neubauer Chamber, and plotted. (**B**) Real-time PCR analysis was used to determine the expression levels of genes related to stemness. ASCs were treated with 5 mM metformin for 72 h, and the transcription levels were used to determine the expression levels of, *BMP7, DPP4, SOX2, Oct3/4, Wnt2, ICAM1, CD90,* and *DLK1*. Representative graphs are of three independent replicates (*n* = 3). * *p* < 0.05, ** *p* < 0.01, *** *p* < 0.001 and **** *p* < 0.001 as compared with control.

Higher differentiation and proliferation rates result in loss of stemness. Since we observed that metformin reduces adipogenic differentiation, we were interested in investigating the effect of metformin treatment on the proliferation of ASCs in vitro. ASCs were treated with metformin, and the cells were counted at day 4 post-culture in the presence or absence of metformin. From our results, we observed that metformin significantly decreases the cell proliferation rate compared to the control ASCs (Figure 2A). Stem cells play an important role in tissue regeneration and maintenance. To efficiently perform their regenerative role, stem cells need to maintain their stemness characteristic. We analyzed the effect of metformin treatment on the expression of stemness genes in ASCs. To this end, we used quantitative real-time PCR to analyze genes related to stemness maintenance in ASCs. We observed that metformin significantly upregulated the expression of adipose stem cell markers, such as *BMP7, DPP4, Sox2, Oct3/4, Wnt2, ICAM1, CD90,* and *DLK1,* compared with the control ASCs (Figure 2B). Our results demonstrate that metformin treatment effectively slows down ASCs proliferation, thus preventing the cells from proliferation exhaustion by enhancing the expression of stemness signature genes.

**Figure 3:**
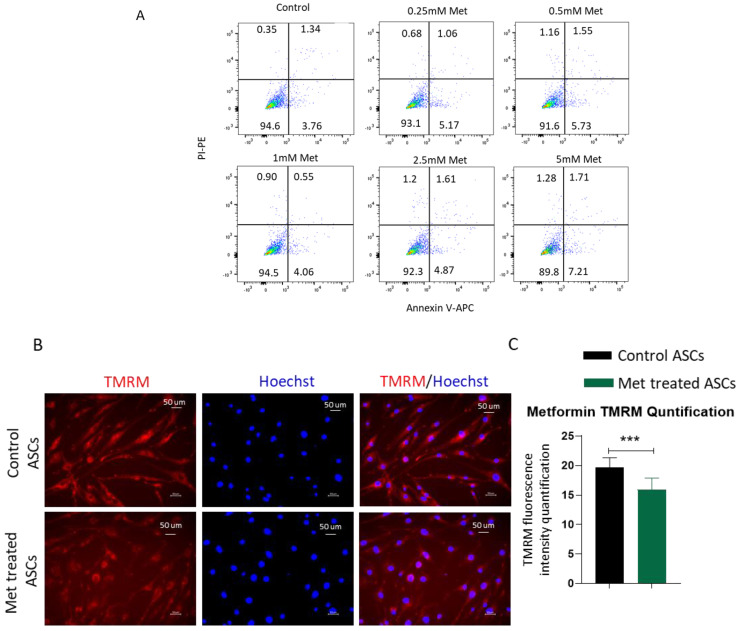

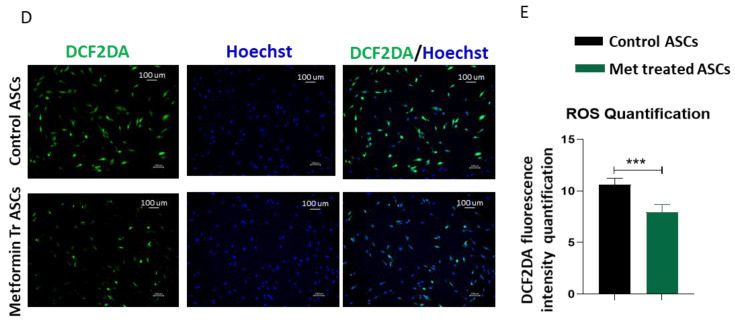
ASCs treated with metformin demonstrate reduced mitochondrial membrane potential and ROS production. (**A**) Adipose stem cells treated with different doses of metformin (0.25, 0.5, 1, 2.5, and 5 Mm) for 72 h showed no induction of apoptosis. ASCs were stained with APC-labeled Annexin V and PI and analyzed by flow cytometry. Representative flow cytometric dot plots of ASCs treated with different doses of metformin. Dot plots represent three independent replicates (*n* = 3). After 72 h of treatment with metformin, ASCs were stained with TMRM or DCF2DA. Representative microscopic image of TMRM- (**B**) or DCF2DA-stained ASCs (**D**). Hoechst was used to stain nuclei. Quantitative analysis of images using image J revealed metformin treatment significantly lowers the mitochondrial membrane potential (**C**) and ROS generation (**E**) in ASCs. Images are representatives of three independent replicates (*n* = 3). *** *p* < 0.001 as compared with control.

To exclude that the decrease in differentiation and proliferation is not due to any cytotoxic effects of metformin on ASCs, we analyzed the viability of metformin-treated cells. Analyses of AnnexinV/PI staining by flow cytometry demonstrated no significant increase in apoptotic or necrotic cells upon treatment with increasing concentrations of metformin up to 5 mM compared to the cell culture medium control (Figure 3A). Mitochondrial activity and the generation of reactive oxygen species (ROS) are important for cell differentiation, and increased levels of mitochondrial metabolism and ROS generation have been observed during adipogenic differentiation [32]. From our results, we observed that metformin treatment reduces the adipogenic differentiation of ASCs. We next investigated the effects on mitochondrial activity and ROS production. ASCs were treated with metformin and stained with TMRM to evaluate mitochondrial membrane activity and with DCF2DA for estimating ROS concentration. Fluorescent microscope images and quantification of the fluorescent signal revealed that metformin treatment significantly reduces mitochondrial activity (Figure 3B,C) and ROS production (Figure 3D,E). We conclude that a metformin-mediated decrease in mitochondrial activity and ROS production contributes to an increase in the stemness of ASCs.

**Figure 4:**
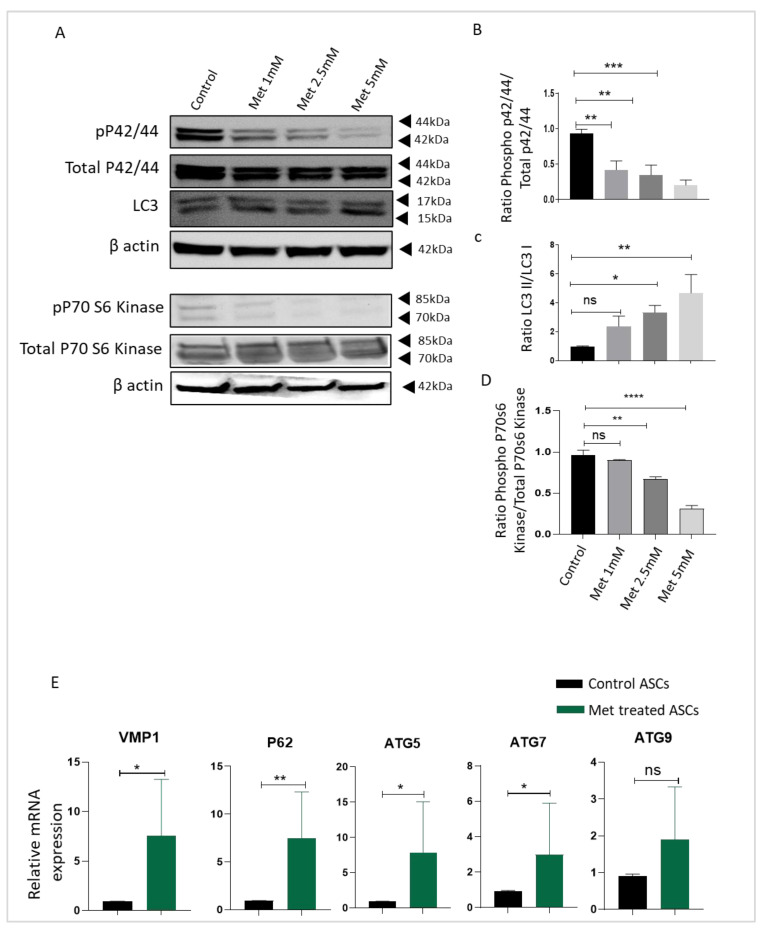
Metformin treatment reduces ERK and mTOR pathway activity and increases autophagy in ASCs. (**A**) Cells were cultured in growth medium containing 0, 1, 2.5, or 5 mM of metformin for 72 h to examine pERK, pP70S6K, and LC3 activity. Results were quantified using densitometry analysis of protein bands and normalized to total ERKs (**B**), LC3 (**C**), or total P70S6K (**D**), accordingly (*n* = 3). Data represent the means ± SEM from three experiments. (**E**) Expression of autophagy reporter genes *ATG5, ATG7, ATG9, VMP1,* and *P62* was determined by real-time quantitative PCR. * *p* < 0.05 and ** *p* < 0.01 as compared with control. * *p* < 0.05, ** *p* < 0.01, *** *p* < 0.001 and **** *p* < 0.0001 as compared with control.

To understand the possible signaling cascade responsible for reduced adipocyte differentiation, proliferation, and upregulation of stemness genes in ASCs upon metformin treatment, we focused on mTOR, autophagy, and extracellular signal-regulated kinase (ERK) signaling cascades. Previous studies have shown the important role of these pathways in maintaining the stemness in ASCs [19,33]. Both ERK and mTOR signaling play an important role in promoting cell proliferation and differentiation. After ASCs were treated with different doses of metformin, cell lysates were collected and Western blotted for LC3, phosphorylated-p70S6 kinase, total p70S6 kinase, phosphorylated-ERK42/44, and total ERK p42/44 antibodies. Beta-actin was employed as a housekeeping gene. Western blot analysis showed that compared with the control ASCs, metformin treatment down-regulated the expression of phosphorylated p70S6K and phosphorylated-ERK42/44 kinase (Figure 4A,B,D). Both ERK and mTOR signaling play an important role in the regulation of the autophagy pathway. Western blot results revealed that metformin treatment enhances autophagy activity in ASCs (Figure 4A,C). We further analyzed the expression of autophagy pathway genes upon metformin treatment at the mRNA level by employing quantitative real-time PCR. With qPCR analysis, we observed a significant increase in the autophagy pathway genes *VMP1, P62, ATG5,* and *ATG7* upon metformin treatment (Figure 4E), which is consistent with the increase in autophagy activity shown in our Western blot results. A non-significant increase in the expression of *ATG 9* was also observed in our analyses. We conclude that metformin treatment reduces ERK and mTOR activity and enhances autophagy in ASCs as a possible underlying mechanism for reduced proliferation and differentiation and increased stemness.

**Figure 5:**
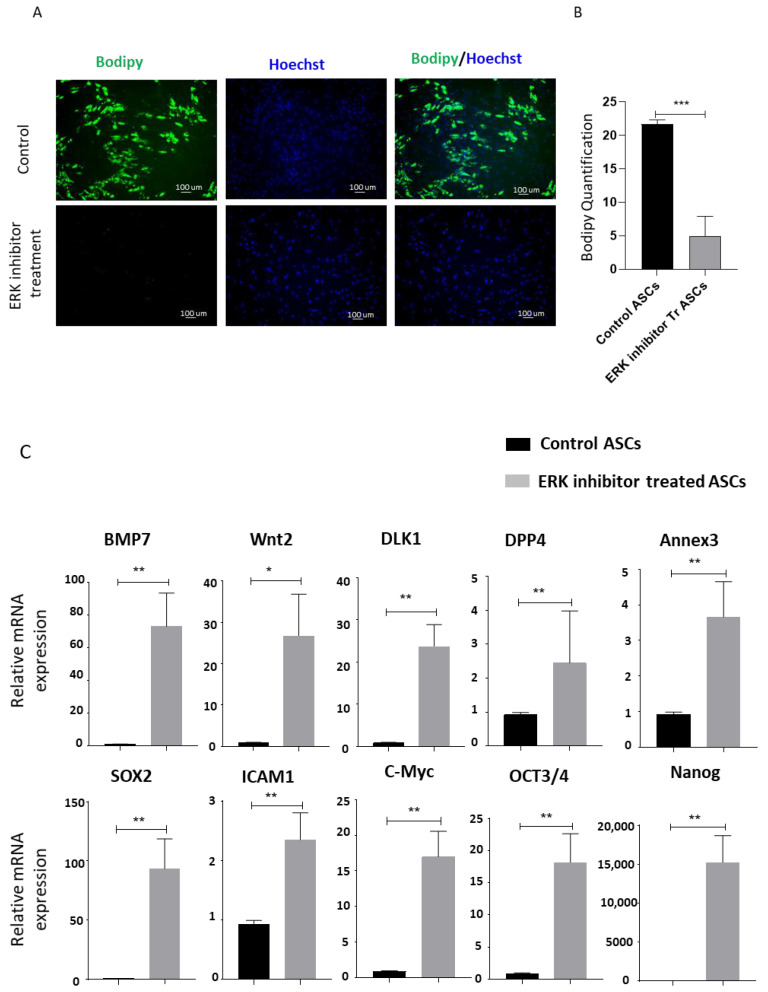
ERK inhibitor treatment inhibits adipogenic differentiation of ASCs. (**A**) ASCs were treated with an adipocyte differentiation cocktail in the presence or absence of 10 µM ERK inhibitor (U0126) for 14 days. Lipid contents on the 14th day were determined by Bodipy stain. Hoechst was used to stain nuclei. (**B**) The fluorescent intensity of Bodipy was determined. Quantification plots of the images were generated using Image J. Images are representatives of three independent replicates (*n* = 3). (**C**) Real-time PCR analysis of gene expression related to the stemness: *BMP7, Wnt 2, DLK1, DPP4, Annex3, SOX2, ICAM1, C-MYC, Oct3/4*, and *Nanog*. * *p* < 0.05, ** *p* < 0.01 and *** *p* < 0.001 as compared with control.

To underscore the direct role of ERK and mTOR signaling in metformin-treatment-mediated inhibition of differentiation and increase in stemness in ASCs, we separately inhibited ERK signaling and the mTOR pathway using ERK inhibitor U0126 and rapamycin, respectively, and analyzed the effects on ASC differentiation and stemness [19,33]. The addition of ERK inhibitor U0126 during adipocyte differentiation of ASCs resulted in significantly reduced differentiation, as revealed by Bodipy stained images and fluorescence quantification (Figure 5A,B). Quantitative real-time PCR results showing an upregulation of stemness genes upon inhibitor-mediated specific inhibition of ERK signaling (Figure 5C) further supported our finding that ERK pathway downregulation by metformin treatment contributes to increasing stemness.

**Figure 6:**
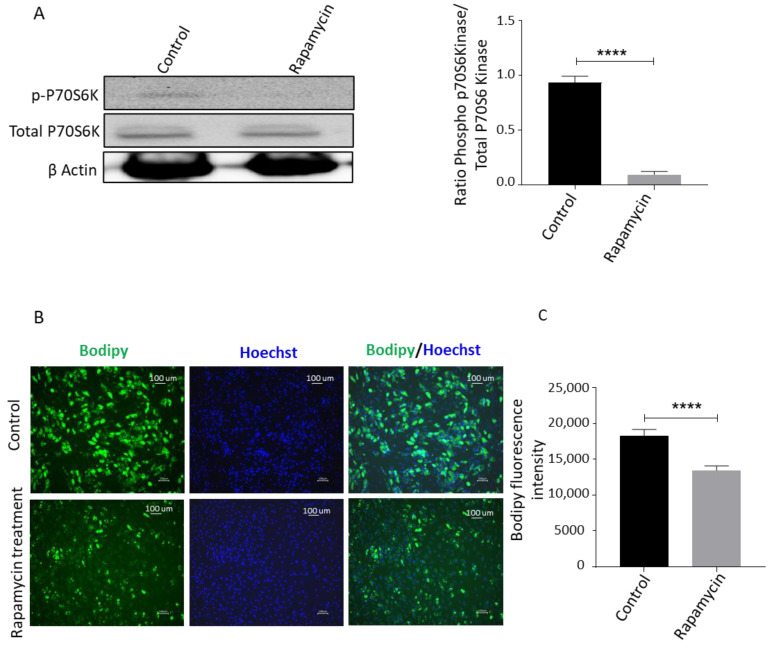

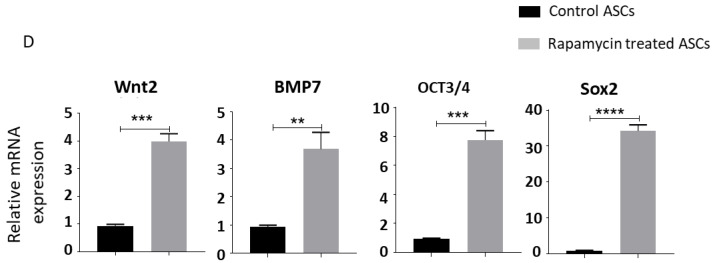
Rapamycin treatment inhibits ASCs adipogenic differentiation and promotes stemness. (**A**) Cell lysates from ASCs treated with 100 nM rapamycin for 72 h were Western blotted for phospho P70S6K, total P70S6K, and β-Actin. Band densities were quantified using Image J software and plotted. (**B**) ASCs were treated with an adipocyte differentiation cocktail in the presence or absence of 100 nM rapamycin for 14 days. The lipid content on the 14th day was determined by Bodipy staining and quantified using Image J (**C**). The data represents the means from 3 independent experiments (*n* = 3). (**D**) Real-time PCR analysis of gene expression related to the stemness: *Wnt2, BMP7, Oct3/4,* and *Sox2*. ** *p* < 0.01, *** *p* < 0.001 and **** *p* < 0.0001 as compared with control.

We then used rapamycin, a well-known mTOR pathway inhibitor that can activate autophagy, and evaluated the effects on adipogenesis and stemness gene expression [19]. Cell lysates of ASCs treated with rapamycin were collected and probed with phosphorylated-p70S6 kinase, total p70S6 kinase, and β-actin antibodies. We observed that rapamycin downregulated the expression of the phosphorylated-p70S6 kinase (Figure 6A). To determine the functional role in adipogenic differentiation, ASCs were treated with rapamycin and induced with the adipogenic medium. After 14 days, the cells were stained with Bodipy stain to assess adipogenesis. We observed that rapamycin treatment significantly reduced adipogenic differentiation (Figure 6B,C). Next, we analyzed the effect of blocking mTOR signal transduction on the stemness of ASCs. To this end, ASCs were treated with rapamycin, and real-time PCR was used to measure the expression of transcripts related to stemness. We observed that rapamycin significantly upregulated the expression of stemness-related markers (Figure 6D). We conclude that inhibition of both ERK and mTOR pathways contributes to a decrease in adipocyte differentiation and increase in stemness in human ASCs.

**Figure 7:**
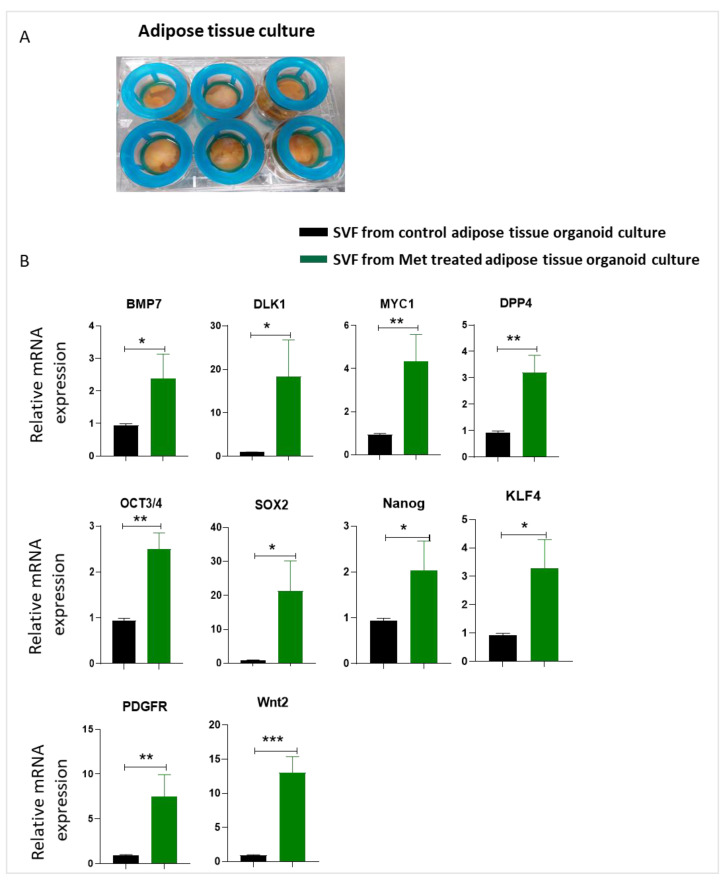
Metformin induces stemness-related gene expression in adipose tissue organoid culture model. (**A**) The adipose tissue was cultured under cell strainer in 6-well plates in a growth medium containing 5 mM metformin for 7 days and then analyzed for expression of stemness-related genes. (**B**) Stromal vascular fraction (SVF) was isolated, and RNA was collected from control and metformin-treated SVFs. The expression levels of *BMP7, DLK1, MYC1, DPP4, Oct3/4, SOX2, Nanog*, *KLF4, PDGFR,* and *Wnt2* were determined. * *p* < 0.05, ** *p* < 0.01, and *** *p* < 0.001 as compared with control.

Next, we tested whether we could reproduce the stemness-enhancing effects of metformin treatment on 2D cultured ASCs in a more complex and in vivo translatable whole adipose tissue culture model. For this, we modified the adipose tissue culture protocol published by Harms et al. [30] using cell strainers instead of transwell inserts to keep the adipose tissue fragments submerged in media (Figure 7A). After culturing 3–5 mm fragments of human adipose tissue with or without metformin for 5 days, we isolated the stromal vascular fraction (SVF) by collagenase digestion of adipose tissue fragments and analyzed the expression of stemness-related genes. Real-time PCR data showed that SVF from adipose tissue treated with metformin demonstrated a significant upregulation in the expression of stemness markers like *BMP7, DLK1, MYC1, DPP4, OCT3/4, Sox2, Nanog, KLF4, PDGFR,* and *Wnt2* (Figure 7B).

## 4. Discussion

Adipose stem cells (ASCs) regenerate adipose tissue through self-renewal and differentiation, thus maintaining the tissue’s stem cell pool. However, aging and obesity are associated with the loss of stemness properties in ASCs. Metformin is an antidiabetic drug and has shown promising results as an anti-aging agent. We focused on analyzing the effect of metformin on the stemness of ASCs and understanding the underlying mechanism, with a goal to establish metformin as a potential agent for maintaining stemness in ASCs with age. For easier translation of our results to human subjects, we employed a 2D cell culture model of human ASCs and used the human adipose tissue culture method to simulate the more complex tissue environment. We used subcutaneous adipose tissue as the tissue source for our studies. Using these diverse cells and tissue culture methods, we studied the effect of metformin treatment on the stemness of ASCs.

Obesity causes an alteration in ASC differentiation kinetics, which decreases the stem cell pool. Our results suggested that, compared with untreated ASCs, metformin treatment reduced adipogenesis rate in a dose-dependent manner. This observation is consistent with earlier reports that showed that metformin inhibits adipogenesis in adipose stem cells, bone marrow stromal cells, periodontal ligament stem cells, and 3T3 cell line [34,35,36,37]. The proliferation rate of stem cells plays an important role in maintaining the stem cell pool, stemness, and self-renewal capability. We observed that metformin inhibited the proliferation of ASCs without inducing any cytotoxic effect on ASCs, which agrees with other studies published on mice adipose-derived stem cells [28], stromal cells, and fibroblasts [38,39]. A recent study using adipose-derived stem cells from horses showed a pro-proliferative effect of metformin [27]. The most likely explanation for these contradictory observations is the difference in the donor species and the anatomical site of cell harvest, as horse ASCs were harvested from the tail. Agnieszka Śmieszek et al. observed that metformin inhibits cell proliferation and promotes cell death with increasing doses (5 and 10 mM) in mouse bone marrow–derived stromal cells and the Balb/3T3 embryonic fibroblast cell line [40]. In addition, metformin inhibits the proliferation of satellite cells isolated from mouse muscles [41]. Supporting our observation, Min-Jung Park et al. reported that metformin improves the anti-inflammatory property of human ASCs by decreasing the expression levels of IL-1, IL-6, and TGF-β; metformin also increases the survival of transplanted ASCs and protects against apoptotic cell death [42]. These results indicate varying effects of metformin treatment on the viability of cells derived from different species and that human ASCs are resistant to the potential cytotoxic effects of metformin treatment.

Differentiation of ASCs into adipocytes is associated with higher energy requirements and metabolic rates, resulting in higher mitochondrial activity and ROS production. ROS plays a critical role in maintaining quiescence and stemness in adult stem cells, as higher levels of ROS push the cells towards differentiation and ultimately stem cell senescence and cell death [43]. We observed that metformin significantly decreases the mitochondrial membrane potential and reactive oxygen species generation. In line with our data, earlier studies also reported that metformin decreases oxidative stress and improves the antioxidant expression in mouse embryonic fibroblasts [44], mouse ASCs [38], and human aortic endothelial cells [45]. Moreover, in agreement with our study, Chien-Hung Lin et al. reported that metformin acts as a neuroprotective agent by reducing oxidative stress in human neural stem cells [46]. Several other studies confirm that metformin acts as an antioxidant by reducing oxidative stress and increasing antioxidant enzyme expression, such as superoxide dismutase in mouse ASCs, murine C2C12 myoblasts, and circulating human endothelial progenitor cells [38,47,48]. Moreover, the study of Alejandro Martin-Montalvo et al. supports our observation, demonstrating that metformin significantly inhibits the mitochondrial complex, inhibiting mitochondrial respiration in mice [49] The effects of metformin on reduced reactive oxygen species generation are possibly mediated by the increase in reduced glutathione [50] in a mechanism involving the redox-sensitive transcription factor Nrf2 [51].

Metformin has been shown to promote the quiescence state of satellite cells by lowering the metabolism required for proliferation and differentiation. Various studies also confirm that metformin strongly promotes the stemness and rejuvenation of stem cells [23,39,41,49,52,53]. It is also known to improve significantly and upregulate the expression levels of stem cell markers. Since we observed that metformin inhibits proliferation and adipogenesis by inhibiting oxidative stress and mitochondrial activity, we sought to confirm whether metformin improves the stemness in ASCs or in an in vitro adipose tissue culture model. Accordingly, we observed that metformin significantly improved the stemness-related marker expression of DPP4, Wnt2, THP1, DLK1, PDGFR, ICAM1, Annexin3, Oct3/4, Nanog, BMP4, SOX2, and Myc1 in 2D ASC culture and adipose tissue culture models. The use of whole adipose tissue culture offers a unique opportunity to simulate in vivo human tissue microenvironment, where the cells are orientated as in their natural architecture and have interactive support from different cell types woven in the tissue matrix. To the best of our knowledge, our study demonstrates for the first time a stemness-enhancing effect of metformin treatment on human ASCs, both in 2D and whole adipose tissue cultures. We consider our results showing an upregulation of stemness genes signature in ASCs isolated from metformin-treated adipose tissue fragments as advocating for further investigation of the use of metformin as a therapy to slow the aging process and rejuvenate aged adipose tissues.

The mechanistic target of rapamycin (mTOR) plays a key role in the proliferation and differentiation of stem cells. Downmodulation of mTOR activity by genetic manipulation, therapeutic intervention, or lifestyle-related changes increases stemness and longevity [19,20]. Since we observed that metformin inhibits adipogenesis, we tested the effect of metformin on mTOR signaling. Interestingly, results showed that metformin downregulates phosphorylation levels of PS70S6 kinase significantly, which is a key signaling molecule downstream of mTOR and a reporter of mTOR pathway activity. Earlier studies also suggest that metformin suppresses adipogenesis by inhibiting the mTOR signaling pathway in mouse embryonic fibroblasts (MEFs), C3H10T1/2 mouse mesenchymal stem cells, and 3T3-L1 preadipocytes [34]. The mechanistic target of rapamycin is composed of two complexes, mTORC1 and mTORC2, and the signaling plays an important role in adipogenesis. Inhibition of mTORC1 signaling with rapamycin is shown to impair adipogenesis via a decrease in phosphorylation of PS6kinase in 3T3-L1 preadipocytes. These studies also demonstrated that inhibition of mTOR signaling inhibits adipogenesis by downregulating the expression levels of adipocyte lineage markers such as *PPARgamma, CEBP1α, CEBP1β*, and *adiponectin* [54,55,56]. Moreover, mTOR signaling is mediated by the phosphorylation of S6 kinase. The knockdown of S6 kinase was shown to be resistant to obesity and weight gain during high-fat diet conditions due to impairment of adipogenesis [57]. We confirmed our observation of metformin-mediated downregulation of mTOR activity as the possible mediator of increase in stemness and reduced differentiation of ASCs by using rapamycin, which specifically blocks mTOR pathway activity.

In addition to mTOR, the ERK signaling pathway plays a crucial role in proliferation and adipogenesis. Xiaomin Ning et al., in their publication, posit that adipogenesis is activated by ERK signaling [58]. Since we observed that metformin inhibited adipogenesis and proliferation, we decided to investigate the effect of metformin on ERK signaling. Consistent with earlier reports, our results also suggested that metformin might be inhibiting adipogenesis via suppression of ERK signaling with an increase in stemness-related gene expression. mTOR signaling is required for cell proliferation and differentiation and is a negative regulator of autophagy [18,19,20]. However, loss of autophagy is linked with loss of stemness, although metformin treatment improved the stemness-related gene expression via activation of autophagy-related marker expression such as LC3II. Thus, our results suggest that metformin impairs adipogenesis by improving stemness in human ASC or human adipose tissue culture models via activating autophagy and inhibiting mTOR signaling pathways. Future in vivo animal and human investigational studies focused on assessing the effects of metformin on the biology of ASCs will guide the adaptation of metformin use as a stem cell anti-aging agent.
